# Biosecurity measures associated with reduced avian influenza infection in poultry settings: a systematic review and meta-analysis with One Health implications

**DOI:** 10.1016/j.onehlt.2026.101638

**Published:** 2026-11-03

**Authors:** Dimitra V. Pouliopoulou, Victoria D’Alessandro, Rachel Watson, Marjan Saeedi, Nicole Billias, Joy MacDermid, Erin Miller, Heather McClinchey, Kieran L. Quinn, Saverio Stranges, Scott Weese, Elena Cini, Eleni Boutsikari, Tiago V. Pereira, Jennie Johnstone, Lauren E. Cipriano, David Fisman, Jessica P. Hopkins, Pavlos Bobos

**Affiliations:** a School of Physical Therapy, University of Western Ontario, London, Ontario, Canada; b Collaborative Specialization in Musculoskeletal Health Research, Bone and Joint Institute, University of Western Ontario, Canada; c Ontario Ministry of Health, Toronto, Ontario, Canada; d Dalla Lana School of Public Health, Institute of Health Policy, Management and Evaluation, Department of Medicine, Sinai Health, University of Toronto, Toronto, Ontario, Canada; e Department of Epidemiology and Biostatistics, Schulich School of Medicine and Dentistry, University of Western Ontario, London, Ontario, Canada; f Department of Pathobiology, Ontario Veterinary College, University of Guelph, Guelph, Ontario, Canada; g Global Sustainability Institute, Faculty of Science and Engineering, Anglia Ruskin University, Cambridge, United Kingdom; h Department of Epidemiology and Biostatistics, Faculty of Medicine, National and Kapodistrian University of Athens, Athens, Greece; i Nuffield Department of Population Health, University of Oxford, Oxford, United Kingdom; j Department of Medicine, University of Toronto, Toronto, Ontario, Canada; k Ivey Business School, University of Western Ontario, London, Ontario, Canada; l Dalla Lana School of Public Health, Division of Epidemiology, University of Toronto, Toronto, Ontario, Canada; m Public Health Ontario, Toronto, Ontario, Canada; n Department of Medicine, Schulich School of Medicine and Dentistry, University of Western Ontario, London, Ontario, Canada; o Department of Health Research Methods, Evidence, and Impact, McMaster University, Hamilton, Ontario, Canada

**Keywords:** Avian influenza, Biosecurity, Infection prevention and control, meta-analysis, One Health

## Abstract

**Background:**

High pathogenicity avian influenza continues to cause large epizootics in poultry and other animal populations, with ongoing concern about zoonotic spillover. We aimed to estimate the association between specific hygiene, personal protective equipment, and biosecurity practices and farm-level avian influenza infection in commercial poultry farms and backyard poultry settings.

**Methods:**

We conducted a systematic review and meta-analysis. MEDLINE, Embase, Scopus, CINAHL, CENTRAL, CABI, Web of Science, and ClinicalTrials.gov were searched from inception to Dec 11, 2025. Eligible studies were empirical studies that compared use versus non-use, or different adherence levels, of hygiene, personal protective equipment, or biosecurity practices and reported farm-level avian influenza infection status. Screening, data extraction and risk of bias assessment was completed by 2 independent reviewers. The main outcome was farm-level avian influenza infection. Odds ratios were pooled using random-effects models. The protocol was registered with PROSPERO, CRD420251174630.

**Findings:**

The search identified 39 observational studies, including 28 case-control, nine cross-sectional, and two prospective cohort studies. Reduced odds of infection were observed for disinfection of equipment and premises overall (OR 0.37, 95% CI 0.14–0.99), human entry/exit decontamination stations (0.61, 0.40–0.94), personal hygiene practices (0.52, 0.35–0.76), contact-pathway PPE (0.67, 0.51–0.87), quarantine/cross-farm contamination control (0.49, 0.34–0.71), and biosecurity information resources (0.26, 0.07–0.91). Overall risk of bias was high in eight studies, some concerns in 24, and low in seven. Between-study heterogeneity varied across analyses. Certainty of evidence ranged from low to moderate.

**Interpretation:**

Practical, contact-focused biosecurity practices were most consistently associated with lower farm-level avian influenza infection odds. Although the observational evidence does not establish causal effectiveness and may be affected by residual confounding, the findings support prioritising feasible measures that interrupt direct transmission pathways while accounting for implementation quality, local context, and behavioural feasibility.

## 1 Introduction

Avian influenza is widely distributed in wild and domestic birds and is classified into low pathogenicity (LPAI) and high pathogenicity (HPAI) according to their virulence in poultry [1]. LPAI viruses generally cause mild or subclinical disease but can serve as precursors for forms of high pathogenicity, including some H5 and H7 strains, through mutation or reassortment events [2]. By contrast, HPAI viruses cause devastating outbreaks with rapid mortality, extensive culling, and severe economic impact [3].

Human infections with avian influenza remain relatively rare but are frequently severe. Since 2003, more than 960 confirmed cases of H5N1 have been reported to the World Health Organization, with case fatality exceeding 50% [4], [5]. Transmission occurs primarily through direct contact with infected poultry, infected mammals, or contaminated environments [6] and although limited instances of human-to-human spread have been suspected, sustained transmission has not been demonstrated [7], [8], [9]. The ongoing potential for zoonotic spillover remains a central concern for pandemic preparedness [10].

Farm and backyard poultry settings are especially challenging because people have close contact with birds, contaminated environments, and shared equipment. Evidence shows that biosecurity measures such as restricted access, clothing and footwear changes, and routine disinfection are associated with lower outbreak risk [11], while modelling suggests that early, strict hygiene and movement controls reduce epidemic size and duration [12]. WHO and CDC guidelines also recommend gloves, respiratory and eye protection, protective clothing, and hand hygiene when handling poultry or contaminated materials [13]. However, PPE and hygiene practices may be difficult to sustain in farm environments because wet, slippery, cold, hot, or windy conditions can affect feasibility, while PPE may impair vision or dexterity and create safety risks during animal handling or equipment use [14].

Empirical evidence on the protective effect of PPE is limited. A recent systematic review assessed farm-level protective practices associated with reduced odds of infection but did not provide pooled estimates of protective interventions [6]. Outbreak investigations suggest that farm workers consistently using PPE are less likely to become infected, though adherence and reporting are inconsistent [15].

The present systematic review and meta-analysis address this gap by evaluating the association between biosecurity measures, hygiene practices and PPE and avian influenza transmission control in poultry farms and backyard poultry settings. Specifically, we aim to assess the association between the use of PPE and hygiene/control practices and the reduction of animal-to-animal transmission of avian influenza (including via intermediary animals or vectors or environmental contamination).

## 2 Methods

### 2.1 Study design

This systematic review with meta-analysis follows the Preferred Reporting Items for Systematic Reviews and Meta-Analyses (PRISMA). The protocol was registered with the PROSPERO number CRD420251174630.

### 2.2 Search strategy and selection criteria

A comprehensive search strategy was developed with a research librarian. We searched MEDLINE (Ovid), Embase (Ovid), Scopus, CINAHL (EBSCO), CENTRAL, CABI, Web of Science and ClinicalTrials.gov from inception to December 11th 2025 without language restrictions. We also conducted a manual search on the reference lists of the included studies for additional studies that met our inclusion criteria. The search strategy included terms related to PPE, biosecurity, infection control and avian influenza. The full search strategy is available in eTable 1 in the Supplement.

Eligibility criteria were defined according to the Population, Exposure, Comparator, and Outcome (PECO) framework. The population comprised commercial poultry farms and backyard poultry settings. Eligible exposures included the use, non-use, or level of compliance with hygiene, biosecurity, and PPE-related practices, including decontamination stations, quarantine measures, information resources, waste-management practices, and other farm-level biosecurity measures. Because these measures differ in their biological mechanisms and operational implementation, they were prespecified and synthesised as distinct exposure categories rather than treated as a single intervention. Comparators were farms or poultry premises in which the relevant practice was absent, used less consistently, or implemented differently. The outcome was avian influenza infection or outbreak status at the farm or poultry-premises level. We included empirical observational studies in which the farm or poultry premises was the epidemiological unit of analysis.

We exported all identified studies to Covidence [16] to remove duplicates. Three independent reviewers (DVP, VD and RW) screened titles and abstracts for eligibility. All discrepancies were carried over to full text screening. Full texts of potentially relevant studies were assessed independently by the same reviewers and discrepancies were resolved through consensus.

### 2.3 Data analysis

A standardized data extraction form was used to collect information from the included studies. Information extracted included study characteristics (author, publication year, study type, outbreak year, setting, country, sample size), avian influenza subtype, epidemiologic unit of measurement, details on the case definitions, sampling procedures and information on the association between hygiene practices and PPE and avian influenza control. One reviewer (DVP) conducted the data extraction, and a second reviewer (VD) cross-verified all extracted data.

The primary outcome was the association between adhering to different hygiene practices and PPE and avian influenza infection control, in poultry farms. For each practice, we extracted the odds ratios comparing farms reporting the use of the specified hygiene practice with those not reporting its use and the corresponding association with avian influenza infection. If both an adjusted and an unadjusted odds ratio were available, we extracted the adjusted value.

Individual biosecurity measures were classified according to the specific practice reported in each study. Measures were first retained at the most detailed level supported by the original report, such as hand washing with soap, use of hand sanitizer, footbaths, changing footwear, glove use, equipment cleaning, or premises disinfection, and were subsequently organised into broader conceptual domains for presentation. Measures were pooled only when the reported practices were considered sufficiently comparable.

We assessed the risk of bias using the Cochrane Risk Of Bias In Non-randomized Studies - of Exposure (ROBINS-E) tool [17]. Two reviewers (DVP, VD) independently assessed the risk of bias, and discrepancies were resolved through consensus. We applied the Grades of Recommendation, Assessment, Development, and Evaluation (GRADE) approach to grade the certainty of the evidence [18], [19], [20], [21], [22], [23], [24].

To summarize odds ratios, we use the inverse-variance random-effects model with the residual maximum likelihood (REML) estimator of the between-study variance and reported summary ORs with a 95% CI. We identified statistical heterogeneity using Cochran's Q test and quantified it using the I^2^ statistic. The between study variance was estimated using tau^2^ [25]. If more than 10 studies were available, we assessed publication bias graphically and small-study effects using Egger's test [26]. We conducted all analyses in Stata 19.5 (StataCorp, College Station, TX, EUA).

Two sensitivity analyses were conducted to account for heterogeneity across the included studies. First, analyses were restricted to studies conducted exclusively in commercial poultry settings to assess whether the findings were robust to differences between commercial and backyard production contexts. A corresponding backyard-only analysis was not conducted because too few studies within the individual biosecurity categories were available to produce meaningful pooled estimates. Second, studies judged to be at high risk of bias were excluded to assess the influence of study quality on the pooled estimates. Additional subgroup analyses by avian influenza subtype or pathotype, poultry species, and production system were not conducted because there were insufficient studies evaluating comparable biosecurity practices within these subgroups to support meaningful and reliable analyses.

## 3 Results

We identified 11,414 references that met search criteria. After removal of duplicates, 5200 studies were screened at the title and abstract level, and 403 underwent full-text review. A total of 39 studies were included in the final analysis (Fig. 1). Reasons for exclusion are summarised in Fig. 1.

**Fig. 1:**
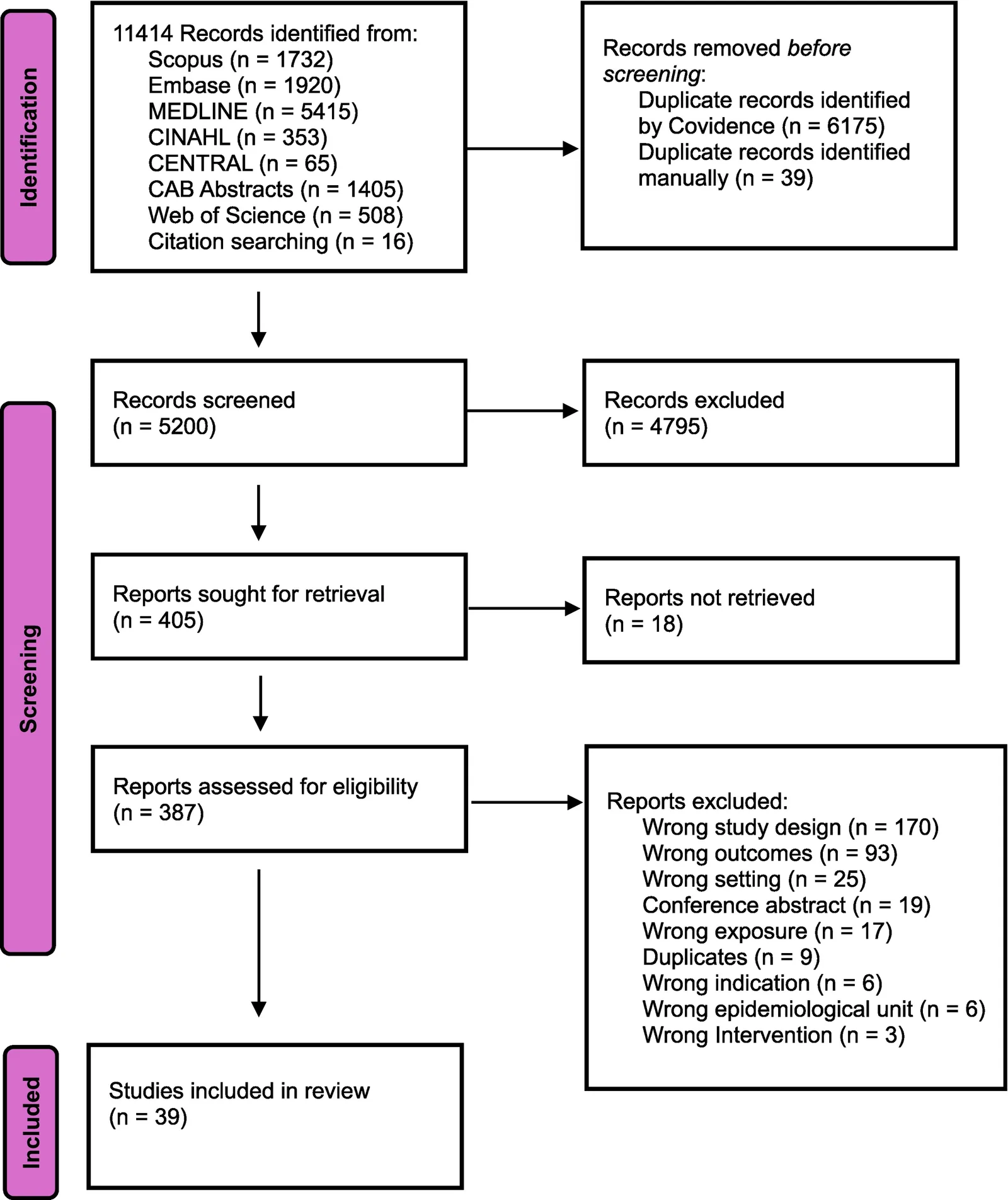
Study Selection - Prisma Flowchart. PRISMA flow diagram showing study identification, screening, eligibility, and inclusion. A total of 11,414 records were identified from database and citation searches: Scopus (*n* = 1732), Embase (*n* = 1920), MEDLINE (*n* = 5415), CINAHL (*n* = 353), CENTRAL (*n* = 65), CAB Abstracts (*n* = 1405), Web of Science (*n* = 508), and citation searching (*n* = 16). After removal of 6214 duplicate records, 5200 records were screened and 4975 were excluded. Of 405 reports sought for retrieval, 2 were not retrieved, leaving 403 reports assessed for eligibility. Reports were excluded for wrong study design, wrong outcome, wrong setting, wrong indication, wrong intervention, duplicates, wrong exposure, conference abstract, or wrong epidemiological unit. Thirty-nine studies were included in the review.

The characteristics of the included studies are summarised in eTable 2 in the Supplement. A total of 39 studies were included [27], [28], [29], [30], [31], [32], [33], [34], [35], [36], [37], [38], [39], [40], [41], [42], [43], [44], [45], [46], [47], [48], [49], [50], [51], [52], [53], [54], [55], [56], [57], [58], [59], [60], [61], [62], [63], [64], [65]. A mapping table linking each extracted exposure variable to the pooled analytical category is presented in eFig. 1.

Twenty seven studies included commercial poultry settings [27], [29], [30], [31], [33], [34], [35], [36], [37], [38], [40], [41], [42], [43], [44], [45], [48], [51], [52], [53], [56], [57], [58], [60], [62], [64], [65], 7 studies included backyard poultry settings [28], [32], [39], [47], [54], [59], [61] and 3 studies [49], [55], [63] included both commercial and backyard settings. Median sample size was 110 farms (interquartile range [IQR]: 83–225) in commercial farms studies and 94 backyard poultry settings (IQR: 65–122) in backyard studies.

Poultry types varied and included chicken-specific [27], [28], [29], [30], [31], [34], [36], [38], [40], [50], [51], [52], [54], [55], [65], turkey-specific [41], [53], [56] and duck-specific production systems [37], [43]. The remaining of the studies included multiple production systems, involving combinations of chickens, ducks, turkeys, waterfowl, quail, ostrich, pigeons, geese, pheasants, ornamental birds, or other poultry types [32], [35], [39], [42], [46], [48], [49], [57], [58], [63], [64]. Some studies reported poultry populations more broadly without specifying a single poultry type [33], [44], [45], [47], [59], [60].

In terms of geographic distribution, 25 studies were conducted in Asia [27], [28], [29], [30], [31], [32], [35], [38], [39], [40], [41], [43], [44], [45], [46], [50], [51], [52], [54], [55], [59], [61], [63], [64], [65], 6 were in North America [34], [36], [48], [53], [56], [62], 6 were in Africa [33], [47], [49], [57], [58], [60] and 2 were in Europe [37], [42].

The majority of the studies investigated H5 subtypes, particularly HPAI H5N1 viruses [27], [28], [29], [33], [36], [39], [45], [47], [49], [52], [53], [54], [60], HPAI H5N2 [34], [62], HPAI H5N8 [37], [43], HPAI H5N6 [65], H5N1 [64], LPAI H5N2 [51], both H5N6 and H5N8 [59] and H5 without specifying the strain [57]. Seven studies investigated H9 strains, including LPAI H9N2 [30], [31], [32], [50], [63], LPAI H9 [55] and H9 without specifying the strain [35]. Three studies investigated both H5 and H9 without further specifying the strain or the pathogenicity [38], [40], [41]. Four studies studied HPAI without specifying the strain [42], [44], [46], [56], and 1 study studied LPAI without specifying the strain [58]. One study investigated LPAI H7N2 [48], and one study investigated both H5, H7 and H9 virus without specifying strains or pathogenicity [61].

Of the 39 included studies, 28 were case-control studies [27], [28], [29], [30], [33], [34], [35], [36], [37], [39], [42], [43], [44], [45], [47], [48], [49], [51], [52], [53], [54], [55], [56], [59], [60], [62], [63], [65], nine were cross-sectional studies [32], [38], [40], [41], [46], [57], [58], [61], [64], and two were prospective cohort studies [31], [50].

For the case-control studies, case farms were most commonly identified through recorded outbreaks or outbreak registries [27], [28], [29], [39], [42], [44], [45], [47], [51], [53], [54], [65] and national or regional surveillance systems [30], [33], [34], [35], [36], [37], [43], [48], [49], [52], [55], [59], [60], [62], [63]. For the cross-sectional studies, two explicitly reported using random sampling approaches [32], [40] and three reported stratified sampling techniques including stratification by turkey density and trading regions [41], by city [61], and by the ratio of poultry breeding units within districts [64]. Thompson et al., [57] included all registered ostrich farms identified. The remaining of the cross-sectional studies did not specify their sampling strategy.

The two cohort studies identified farms through surveillance lists [31], [50]. Chaudry et al., [31] did not confirm non-infection status at baseline, and Mirzaie et al., [50] excluded studies that were tested positive three days post recruitment.

The samples used to confirm infection varied across all study designs. Fourteen studies sampled sick and/or dead birds [27], [29], [30], [31], [39], [40], [43], [48], [49], [50], [52], [54], [55], [59]. Healthy birds were explicitly sampled in one study [38]. One study collected environmental or farm-premises samples, including feces, swabs, drinking water, sewage, cage, and surface samples [61]. Twenty two studies did not specify the health status of sampled birds or the sample used to confirm infection [28], [32], [33], [34], [35], [36], [37], [41], [42], [44], [45], [47], [51], [53], [56], [57], [58], [60], [62], [63], [64], [65] and one relied on self-reported infection status without laboratory confirmation [46].

Diagnostic approaches also varied. PCR-based methods, either alone or combined with other tests, were reported in 22 studies [27], [28], [29], [35], [36], [37], [39], [40], [41], [42], [43], [48], [49], [50], [52], [53], [54], [55], [58], [59], [61], [62]. Serological or antigen-based methods without PCR were reported in seven studies [30], [31], [32], [38], [51], [56], [63]. Nine studies reported unspecified virological, molecular, or laboratory confirmation methods [33], [34], [44], [45], [47], [56], [60], [64], [65], and one study used self-reported infection status without laboratory confirmation [46].

For the case control studies, eighteen studies matched the controls to the cases by geographic area or proximity [27], [28], [29], [33], [34], [35], [36], [37], [39], [43], [45], [47], [48], [49], [51], [52], [56], [59]. Eight studies reported matching by production type, poultry type, or species [27], [29], [37], [39], [42], [48], [59], [62], 4 studies reported matching by flock or farm size [33], [35], [42], [63], and 5 studies reported matching by time of the outbreak. Other matching factors included laboratory of submission [30], distance from coastal areas or wetlands [42], production stage [62], and number of hired employees and flock age [63]. Five case control studies did not report clear matching criteria [44], [54], [56], [60], [65].

### 3.1 Risk of bias

Risk of bias was assessed using ROBINS-E. Based on the overall judgement, eight studies were judged to be at high risk of bias [27], [40], [41], [46], [47], [48], [49], [64], 24 studies were judged to have some concerns [28], [29], [30], [33], [34], [35], [36], [37], [39], [43], [44], [45], [51], [52], [53], [54], [55], [56], [57], [58], [59], [60], [62], [65], and seven studies were judged to be at low risk of bias ([31], [32], [38], [42], [50], [61], [63] (eTable 3, Supplement).

### 3.2 Disinfection of equipment and premises

A total of 15 studies [27], [28], [30], [34], [36], [37], [39], [40], [41], [43], [44], [54], [59], [61], [64] reported on disinfection of equipment and premises as protective measures against avian influenza. Three studies [34], [36], [40] reported on cleaning farm equipment. The pooled estimate indicates that farms that clean their equipment had 72% reduced odds of being positive for avian influenza compared to farms that do not clean their equipment (OR: 0.28; 95% CI: 0.14 to 0.56; 3 studies, 324 farms). The heterogeneity was low (τ^2^: 0.00). The certainty of evidence was rated as moderate, downgraded for high risk of bias (Fig. 2).

**Fig. 2:**
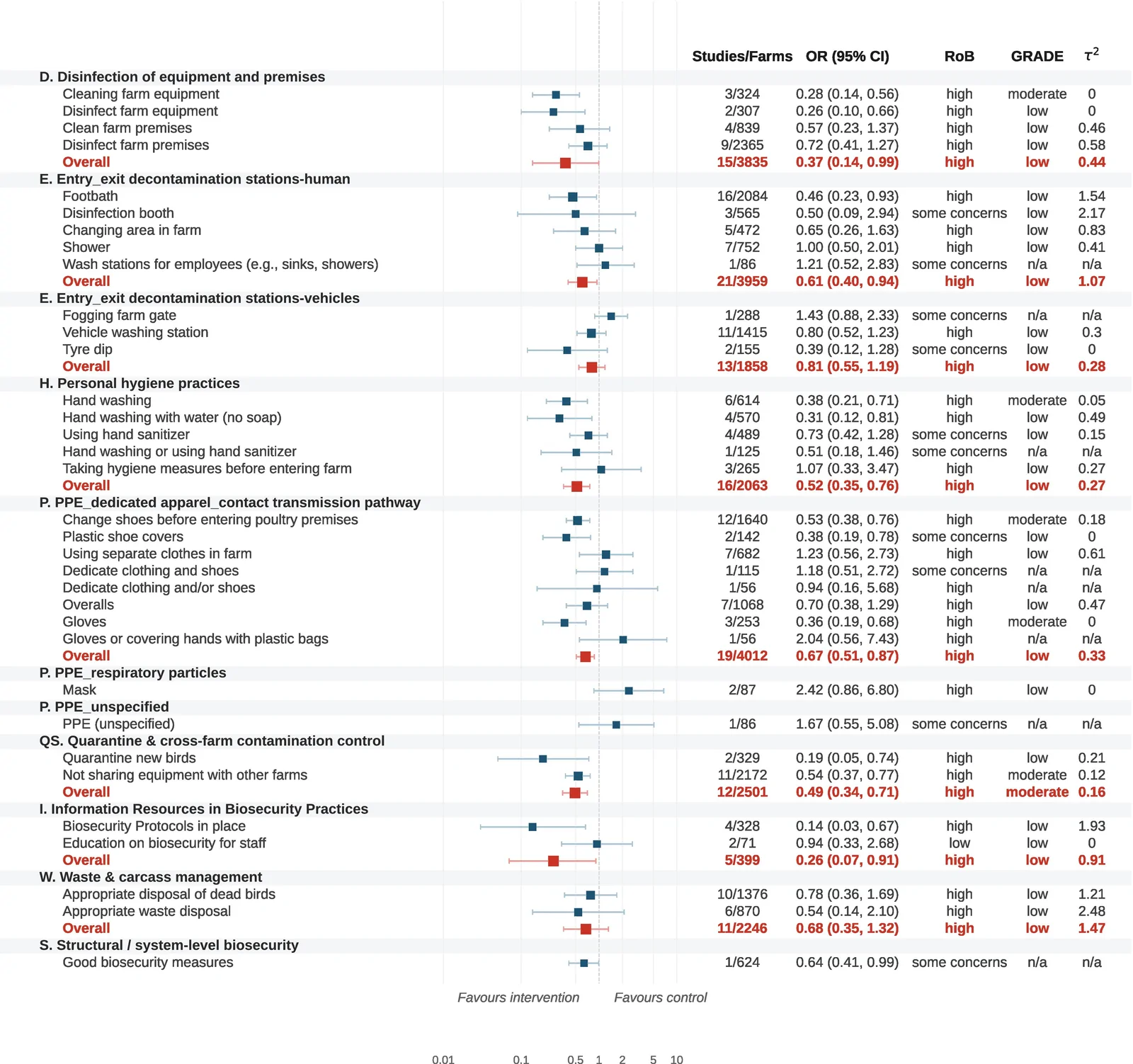
Master Forest plot. Forest plot summarising pooled odds ratios for PPE, hygiene, and biosecurity practices associated with avian influenza infection at the farm level. Outcomes are grouped by intervention category: disinfection of equipment and premises; human entry/exit decontamination; vehicle entry/exit decontamination; personal hygiene; PPE for contact transmission; PPE for respiratory particles; unspecified PPE; quarantine and cross-farm contamination control; information resources in biosecurity practices; waste and carcass management; and structural/system-level biosecurity. For each practice, the figure reports the number of studies and farms, odds ratio with 95% confidence interval, risk of bias, GRADE certainty, and τ^2^. Several practices favoured intervention, including cleaning farm equipment, disinfecting farm equipment, footbaths, hand washing, changing shoes before entering poultry premises, gloves, quarantine of new birds, not sharing equipment between farms, and biosecurity protocols. Overall pooled estimates are shown in red for categories with multiple practices. The x-axis is on a logarithmic scale, with values below 1 favouring the intervention and values above 1 favouring the control.

Two studies [41], [64] reported on disinfection of farm equipment. The pooled estimate indicates that farms that disinfect their equipment had 74% reduced odds of being positive for avian influenza compared to farms that do not disinfect their equipment (OR: 0.26; 95% CI: 0.10 to 0.66; 2 studies, 307 farms). The heterogeneity was low (τ^2^: 0.00). The certainty of evidence was rated as low, downgraded for high risk of bias and imprecision (Fig. 2).

Four studies [28], [37], [59], [61] reported on cleaning farm premises. The pooled estimate indicates that the odds of being positive for avian influenza did not differ between farms that cleaned their premises and those that did not (OR: 0.57; 95% CI: 0.23 to 1.37; 4 studies, 839 farms). The heterogeneity was moderate (τ^2^: 0.46). The certainty of evidence was rated as low, downgraded for high risk of bias and inconsistency (Fig. 2).

Nine studies [27], [28], [30], [39], [43], [44], [54], [59], [61] reported on disinfection of farm premises. The pooled estimate indicates that the odds of being positive for avian influenza did not differ between farms that disinfected their premises and those that did not (OR: 0.72; 95% CI: 0.41 to 1.27; 9 studies, 2365 farms). The heterogeneity was moderate (τ^2^: 0.58). The certainty of evidence was rated as low, downgraded for high risk of bias and inconsistency (Fig. 2).

Overall, the pooled estimate across 15 studies indicates that disinfection of equipment and premises was associated with reduced odds of avian influenza infection (OR: 0.37; 95% CI: 0.14 to 0.99; 15 studies, 3835 farms). The heterogeneity was moderate (τ^2^: 0.44). The certainty of evidence was rated as low, downgraded for high risk of bias and inconsistency (Fig. 2).

### 3.3 Entry and exit decontamination stations for humans

A total of 21 studies [27], [30], [31], [33], [34], [40], [41], [42], [43], [44], [45], [46], [48], [49], [50], [53], [56], [62], [63], [64], [65] reported on entry and exit decontamination stations for humans. Sixteen studies [27], [30], [31], [33], [34], [40], [41], [42], [43], [45], [46], [48], [49], [56], [62], [63] reported on the use of footbaths. The pooled estimate indicates that farms using footbaths had 54% reduced odds of being positive for avian influenza compared to farms that did not use footbaths (OR: 0.46; 95% CI: 0.23 to 0.93; 16 studies, 2084 farms). The heterogeneity was high (τ^2^: 1.54). The certainty of evidence was rated as low, downgraded for high risk of bias and inconsistency (Fig. 2).

Three studies [44], [50], [65] reported on disinfection booths (OR: 0.50; 95% CI: 0.09 to 2.94; 3 studies, 565 farms; τ^2^: 2.17). Five studies [33], [34], [43], [45], [64] reported on changing areas in farms (OR: 0.65; 95% CI: 0.26 to 1.63; 5 studies, 472 farms; τ^2^: 0.83), and seven studies [33], [34], [42], [48], [50], [53], [63] reported on showers (OR: 1.00; 95% CI: 0.50 to 2.01; 7 studies, 752 farms; τ^2^: 0.41). One study [56] reported on wash stations for employees (OR: 1.21; 95% CI: 0.52 to 2.83; 1 study, 86 farms) (Fig. 2).

Overall, the pooled estimate across 21 studies indicates that entry and exit decontamination stations for humans were associated with reduced odds of avian influenza infection (OR: 0.61; 95% CI: 0.40 to 0.94; 21 studies, 3959 farms). The heterogeneity was moderate to high (τ^2^: 1.07). The certainty of evidence was rated as low, downgraded for high risk of bias and inconsistency (Fig. 2).

### 3.4 Entry and exit decontamination stations for vehicles

A total of 13 studies [27], [33], [34], [36], [44], [45], [46], [48], [51], [53], [56], [57], [62] reported on entry and exit decontamination stations for vehicles. Eleven studies [27], [34], [36], [45], [46], [48], [51], [53], [56], [57], [62] reported on vehicle washing stations. The pooled estimate indicates that the odds of being positive for avian influenza did not differ between farms with and without vehicle washing stations (OR: 0.80; 95% CI: 0.52 to 1.23; 11 studies, 1415 farms). The heterogeneity was low (τ^2^: 0.30). The certainty of evidence was rated as low, downgraded for high risk of bias and inconsistency. Two studies [33], [36] reported on tyre dips (OR: 0.39; 95% CI: 0.12 to 1.28; 2 studies, 155 farms; τ^2^: 0.00), and one study [44] reported on fogging farm gates (OR: 1.43; 95% CI: 0.88 to 2.33; 1 study, 288 farms) (Fig. 2).

Overall, the pooled estimate across 13 studies indicates that entry and exit decontamination stations for vehicles were not associated with reduced odds of avian influenza infection (OR: 0.81; 95% CI: 0.55 to 1.19; 13 studies, 1858 farms). The heterogeneity was low (τ^2^: 0.28). The certainty of evidence was rated as low, downgraded for high risk of bias and inconsistency (Fig. 2).

### 3.5 Personal hygiene practices

A total of 14 studies [27], [29], [34], [40], [41], [42], [43], [44], [45], [47], [49], [51], [53], [60] reported on personal hygiene practices. Six studies [34], [40], [41], [42], [47], [60] reported on hand washing. The pooled estimate indicates that farms where hand washing was practiced had 62% reduced odds of being positive for avian influenza compared to farms where it was not practiced (OR: 0.38; 95% CI: 0.21 to 0.71; 6 studies, 614 farms). The heterogeneity was low (τ^2^: 0.05). The certainty of evidence was rated as moderate, downgraded for high risk of bias (Fig. 2).

Four studies [40], [41], [47], [49] reported on hand washing with water only (OR: 0.31; 95% CI: 0.12 to 0.81; 4 studies, 570 farms; τ^2^: 0.49), and four studies [42], [43], [44], [45] reported on use of hand sanitizer (OR: 0.73; 95% CI: 0.42 to 1.28; 4 studies, 489 farms; τ^2^: 0.15). One study [53] reported on hand washing or sanitizer use (OR: 0.51; 95% CI: 0.18 to 1.46; 1 study, 125 farms), and three studies [27], [29], [51] reported on taking hygiene measures before entering farms (OR: 1.07; 95% CI: 0.33 to 3.47; 3 studies, 265 farms; τ^2^: 0.27) (Fig. 2).

Overall, the pooled estimate across 14 studies indicates that personal hygiene practices were associated with reduced odds of avian influenza infection (OR: 0.52; 95% CI: 0.35 to 0.76; 13 studies, 2063 farms). The heterogeneity was low (τ^2^: 0.27). The certainty of evidence was rated as low, downgraded for high risk of bias and inconsistency (Fig. 2).

### 3.6 Personal protective equipment-contact transmission pathway

A total of 16 studies [30], [31], [33], [34], [35], [36], [40], [41], [42], [45], [47], [48], [49], [52], [55], [60], [62], [63], [65] reported on personal protective equipment related to contact transmission pathways. Twelve studies [30], [31], [35], [42], [45], [48], [49], [52], [55], [60], [63], [65] reported on changing shoes before entering poultry premises. The pooled estimate indicates that this practice was associated with reduced odds of avian influenza infection (OR: 0.53; 95% CI: 0.38 to 0.76; 12 studies, 1640 farms). The heterogeneity was low (τ^2^: 0.18). The certainty of evidence was rated as moderate, downgraded for high risk of bias (Fig. 2).

Two studies [34], [62] reported on wearing plastic shoe covers (OR: 0.38; 95% CI: 0.19 to 0.78; 2 studies, 142 farms; τ^2^: 0.00). Seven studies [34], [36], [40], [41], [42], [45], [60] reported on using separate clothes in farms (OR: 1.23; 95% CI: 0.56 to 2.73; 7 studies, 682 farms; τ^2^: 0.61), and seven studies [30], [34], [35], [48], [49], [55], [62] reported on overalls (OR: 0.70; 95% CI: 0.38 to 1.29; 7 studies, 1068 farms; τ^2^: 0.47). Three studies [31], [49], [60] reported on gloves (OR: 0.36; 95% CI: 0.19 to 0.68; 3 studies, 253 farms; τ^2^: 0.00). Single-study estimates were reported for dedicated clothing and shoes [33] (OR: 1.18; 95% CI: 0.51 to 2.72; 1 study, 115 farms), dedicated clothing and/or shoes [47] (OR: 0.94; 95% CI: 0.16 to 5.68; 1 study, 56 farms), and wearing gloves or covering hands with plastic bags [47] (OR: 2.04; 95% CI: 0.56 to 7.43; 1 study, 56 farms) (Fig. 2).

Overall, the pooled estimate across 19 studies indicates that personal protective equipment for contact transmission pathways was associated with reduced odds of avian influenza infection (OR: 0.67; 95% CI: 0.51 to 0.87; 16 studies, 4012 farms). The heterogeneity was low (τ^2^: 0.33). The certainty of evidence was rated as low, downgraded for high risk of bias and inconsistency (Fig. 2).

### 3.7 Personal protective equipment & dedicated apparel - respiratory particles and mixed

Two studies [42], [47] reported on mask use for respiratory protection (OR: 2.42; 95% CI: 0.86 to 6.80; 2 studies, 87 farms). The heterogeneity was low (τ^2^: 0.00). The certainty of evidence was rated as low, downgraded for high risk of bias and imprecision. One study reported on unspecified PPE use [56] (OR: 1.67; 95% CI: 0.55 to 5.08; 1 study, 86 farms) (Fig. 2).

### 3.8 Quarantine and cross-farm contamination control

A total of 12 studies [30], [31], [33], [38], [41], [45], [48], [51], [54], [55], [57], [59] reported on quarantine and cross-farm contamination control. Two studies [41], [59] reported on quarantining new birds. The pooled estimate indicates that this practice was associated with reduced odds of avian influenza infection (OR: 0.19; 95% CI: 0.05 to 0.74; 2 studies, 329 farms). The heterogeneity was low (τ^2^: 0.21). The certainty of evidence was rated as low, downgraded for high risk of bias and inconsistency (Fig. 2).

Eleven studies [30], [31], [33], [38], [41], [45], [48], [51], [54], [55], [57] reported on not sharing equipment with other farms (OR: 0.54; 95% CI: 0.37 to 0.77; 11 studies, 2172 farms; τ^2^: 0.12). The certainty of evidence was rated as moderate, downgraded for high risk of bias (Fig. 2).

Overall, the pooled estimate across 12 studies indicates that quarantine and cross-farm contamination control were associated with reduced odds of avian influenza infection (OR: 0.49; 95% CI: 0.34 to 0.71; 12 studies, 2501 farms). The heterogeneity was low (τ^2^: 0.16). The certainty of evidence was rated as moderate, downgraded for high risk of bias (Fig. 2).

### 3.9 Information resources in biosecurity practices

A total of 5 studies [42], [43], [62], [63], [64] reported on information resources in biosecurity practices. Four studies [42], [43], [62], [64] reported on having biosecurity protocols in place. The pooled estimate indicates that farms with biosecurity protocols had reduced odds of avian influenza infection (OR: 0.14; 95% CI: 0.03 to 0.67; 4 studies, 328 farms). The heterogeneity was high (τ^2^: 1.93). The certainty of evidence was rated as low, downgraded for high risk of bias and inconsistency (Fig. 2).

Two studies [42], [63] reported on education on biosecurity for staff (OR: 0.94; 95% CI: 0.33 to 2.68; 2 studies, 71 farms; τ^2^: 0.00). The certainty of evidence was rated as low, downgraded for high risk of bias and imprecision (Fig. 2).

Overall, the pooled estimate across 5 studies indicates that information resources in biosecurity practices were associated with reduced odds of avian influenza infection (OR: 0.26; 95% CI: 0.07 to 0.91; 5 studies, 399 farms). The heterogeneity was moderate (τ^2^: 0.91). The certainty of evidence was rated as low, downgraded for high risk of bias and inconsistency (Fig. 2).

### 3.10 Waste and carcass management

A total of 11 studies [30], [31], [32], [33], [36], [37], [38], [43], [48], [49], [60] reported on waste and carcass management. Ten studies [30], [32], [33], [36], [37], [38], [43], [48], [49], [60] reported on appropriate disposal of dead birds (OR: 0.78; 95% CI: 0.36 to 1.69; 10 studies, 1376 farms; τ^2^: 1.21), and six studies [31], [33], [38], [43], [48], [49] reported on appropriate waste disposal (OR: 0.54; 95% CI: 0.14 to 2.10; 6 studies, 870 farms; τ^2^: 2.48) (Fig. 2).

Overall, the pooled estimate across 11 studies indicates that waste and carcass management practices were not associated with reduced odds of avian influenza infection (OR: 0.68; 95% CI: 0.35 to 1.32; 11 studies, 2246 farms). The heterogeneity was high (τ^2^: 1.47). The certainty of evidence was rated as low, downgraded for high risk of bias and inconsistency (Fig. 2).

### 3.11 Sensitivity analysis

Two separate sensitivity analyses were conducted to assess the robustness of the primary findings. In the first sensitivity analysis, studies judged to be at high risk of bias were excluded. In the second sensitivity analysis, analyses were restricted to studies conducted exclusively in commercial poultry settings. The results are presented, in eFig 2–19 in the Supplement.

The sensitivity analyses produced no meaningful changes in the overall direction or magnitude of the findings after restricting analyses to commercial farms or excluding studies at high risk of bias. Some associations were no longer statistically significant or had wider confidence intervals, largely reflecting the smaller number of studies and reduced precision rather than substantive changes in the estimates.

## 4 Discussion

This systematic review and meta-analysis synthesised evidence on PPE, hygiene, and farm-level biosecurity practices for reducing avian influenza infection risk in poultry settings. Practices targeting direct contact transmission were most consistently associated with lower odds of infection, including equipment disinfection, footbaths, hand hygiene, changing footwear before entering poultry premises, glove use, not sharing equipment, and quarantining new birds. In contrast, broader environmental or indirect measures, including premises disinfection and vehicle-related decontamination, showed inconsistent or non-significant effects. Masks and other respiratory protective equipment were not associated with reduced odds of farm-level infection, although evidence was limited. Because all studies were observational, findings should be interpreted cautiously given residual confounding, recall bias, social desirability bias, exposure misclassification, and generally low certainty of evidence.

Non-significant findings should not be interpreted as evidence that these measures have no effect. For several practices, estimates were imprecise and the certainty of evidence was low, while variation in exposure definition, implementation quality, study power, and residual confounding may have obscured true associations. These findings therefore indicate insufficient or inconsistent evidence of an association rather than evidence of no benefit.

These findings should be understood within multimodal infection prevention and control. Measures such as dedicated footwear, gloves, equipment segregation, and quarantine likely performed more consistently because they interrupt clear transmission pathways and can be embedded into routine workflows. Environmental and system-level interventions may depend more heavily on correct application, maintenance, infrastructure, and coverage across complex farm environments. Variability in waste and carcass management illustrates this context dependence, as appropriate disposal differs by regional infrastructure, regulations, and environmental conditions. Similarly, education alone did not show a clear protective effect, whereas established biosecurity protocols were associated with reduced odds of infection, suggesting that visible, repeated, and operationalised practices may support sustained behaviour change better than one-off information.

Our findings align with and extend previous evidence. Gkrinia et al. [11] similarly found that biosecurity practices targeting people and equipment were protective in indoor commercial poultry settings but used narrative synthesis only. By providing stratified pooled odds ratios across eight biosecurity domains, this review enables comparison of specific practices. Fournié et al. [66] reported that direct and indirect poultry contact were dominant pathways for human avian influenza infection; our findings complement this by showing that contact-pathway interventions may reduce farm-level infection risk at the animal-human interface. Evidence from the 2024 H5N1 dairy outbreak highlights the difficulty of sustaining PPE adherence in agricultural work, where use may be reactive and constrained by comfort, availability, and workflow [14]. Recent modelling by Gonnerman et al. [67] supports the importance of on-farm biosecurity beyond spatial and environmental risks, while Duarte et al. [68] highlighted heterogeneity in biosecurity definitions and measurement.

This review has several strengths. To our knowledge, it is the first large-scale meta-analysis to quantitatively synthesise specific hygiene, PPE, and biosecurity practices against farm-level avian influenza infection risk. It followed a prospectively registered protocol, searched animal and human health databases, included 39 studies across diverse regions, production systems, and viral subtypes, focused on farms as the epidemiological unit, and used random-effects models, stratified syntheses, sensitivity analyses, and GRADE assessments. However, studies were observational, heterogeneous, and often at some concerns or high risk of bias. Many practices, especially waste management and biosecurity protocols, were poorly defined or variably reported.

Outcome ascertainment varied across studies, and incomplete confirmation of infection status, particularly among comparison farms, may have resulted in misclassification of subclinical or low-pathogenicity infections. This limitation was reflected in the risk-of-bias assessments and examined through the sensitivity analysis excluding studies at high risk of bias.

The included studies varied in avian influenza subtype and pathotype, poultry species, production system, geographic region, and commercial versus backyard setting. These differences may influence transmission pathways, baseline infection risk, and the effectiveness or implementation of individual biosecurity practices. Although sensitivity analyses were conducted for commercial poultry settings and after excluding studies at high risk of bias, the available evidence did not permit meaningful subgroup analyses by HPAI versus LPAI, specific poultry species, or production system.

Although practices were classified and synthesised at the most specific level supported by the included studies, the quality of exposure reporting was variable. Many studies identified whether a practice was present or absent but provided limited information on its frequency, consistency, products used, maintenance, staff adherence, or implementation fidelity. Consequently, farms reporting the same practice may not have implemented it in an equivalent manner, creating a risk of exposure misclassification and potentially contributing to between-study heterogeneity.

Temporality was also uncertain in most included studies. Except for the 2 prospective cohort studies, biosecurity practices were generally assessed retrospectively, and it was not always possible to confirm that they were in place before infection occurred. Some practices may have been introduced or strengthened in response to an outbreak, particularly disinfection procedures, PPE use, footbaths, and formal biosecurity protocols. Reactive adoption among infected farms could attenuate protective associations by increasing the reported use of these practices among cases; however, retrospective reporting, differential recall, and social desirability may introduce bias in either direction.

The pooled estimates should therefore be interpreted as average associations with the reported presence or use of each practice across the settings and viral contexts represented in the included studies, rather than as effects of standardized, uniformly implemented interventions that are necessarily consistent across all poultry populations and avian influenza viruses.

Biosecurity practices were also rarely implemented in isolation, and individual studies often reported several related practices within the same domain. Consequently, the estimated association for one reported practice may partly reflect the broader biosecurity context in which it was implemented. In addition, because some studies contributed estimates to more than one strategy-specific subgroup, the overall domain estimates should be interpreted as descriptive summaries, with greater emphasis placed on the individual strategy-specific results.

The policy implications are practical but implementation dependent. Strategies should prioritise high-impact, behaviourally feasible practices with consistent protective associations, including equipment segregation, quarantine of new birds, dedicated footwear, glove use, and hand hygiene. More resource-intensive measures, such as vehicle decontamination or broad environmental disinfection, may provide lower or more variable return on investment unless implemented with high fidelity. Investment should therefore include training, monitoring, workflow design, and systems that support correct use.

Future studies should use stronger methods to confirm both infection and non-infection status, particularly among comparison farms. Case-control studies should avoid defining controls solely by the absence of reported outbreaks or clinical signs, as this may misclassify farms with undetected or subclinical infection. Cross-sectional studies should use stratified random sampling from clearly defined farm populations and sample a sufficient number of birds per farm to improve the likelihood of detecting infection when present. Cohort studies should confirm non-infection at baseline so that subsequent cases reflect incident infection rather than pre-existing undetected infection.

Importantly, research should move beyond self-report using observation, audits, or sensor-based monitoring, and evaluate effectiveness and cost-effectiveness using pragmatic trials. Prospective studies of high-exposure workers are needed to clarify respiratory protection, and One Health approaches should link farm-level biosecurity to human spillover risk.

## 5 Conclusion

In conclusion, layered biosecurity measures targeting direct contact pathways were most consistently associated with lower odds of farm-level avian influenza infection, while broader environmental measures showed inconsistent associations. Although the observational evidence does not establish causal effectiveness and may be affected by residual confounding, the findings support prioritising practical, low-cost, and reliably implemented interventions within routine farm operations.

## Data sharing

All data used in this systematic review and meta-analysis were extracted from publicly available published studies. The full dataset of the extracted data and software codes are available from the senior author, PB, upon reasonable request.

## CRediT authorship contribution statement

**Dimitra V. Pouliopoulou:** Writing – original draft, Visualization, Project administration, Methodology, Investigation, Formal analysis, Data curation, Conceptualization. **Victoria D’Alessandro:** Writing – review & editing, Investigation, Data curation. **Rachel Watson:** Writing – review & editing, Investigation. **Marjan Saeedi:** Writing – review & editing, Project administration. **Nicole Billias:** Writing – review & editing. **Joy MacDermid:** Writing – review & editing. **Erin Miller:** Writing – review & editing. **Heather McClinchey:** Writing – review & editing. **Kieran L. Quinn:** Writing – review & editing. **Saverio Stranges:** Writing – review & editing. **Scott Weese:** Writing – review & editing, Formal analysis. **Elena Cini:** Writing – review & editing. **Eleni Boutsikari:** Writing – review & editing. **Tiago V. Pereira:** Writing – review & editing, Formal analysis. **Jennie Johnstone:** Writing – review & editing. **Lauren E. Cipriano:** Writing – review & editing. **David Fisman:** Writing – review & editing. **Jessica P. Hopkins:** Writing – review & editing, Formal analysis. **Pavlos Bobos:** Writing – review & editing, Validation, Supervision, Methodology, Funding acquisition, Formal analysis, Conceptualization.

## Funding

This work was supported by the 10.13039/501100000024Canadian Institutes of Health Research (CIHR) Catalyst Grant: Catalyzing OneHealth Research on Avian Influenza (Funding Reference Number: 525182). The funders had no role in the study design, data collection and analysis, decision to publish, or preparation of the manuscript.

## Declaration of competing interest

The authors declare that they have no known competing financial interests or personal relationships that could have appeared to influence the work reported in this paper.

## Data Availability

Data will be made available on request.
