# Supplementary material for: Biosecurity measures associated with reduced avian influenza infection in poultry settings: a systematic review and meta-analysis with One Health implications
Source: One Health. 2026 Nov 3;23:101638. doi: 10.1016/j.onehlt.2026.101638 (PMC13769099; doi:10.1016/j.onehlt.2026.101638)
Supplement: Supplementary file 1 [file mmc1.docx]

**Supplement**

**Title:** Biosecurity measures to prevent Avian Influenza in poultry farms and human spillover: A Systematic Review and Meta-Analysis

Table of Contents

[e Table 1. Search Strategy	2](#_Toc236683593)

[eTable 2. Characteristics of the included studies	3](#_Toc236683594)

[eTable 3. Risk of bias	9](#_Toc236683595)

[eFigure 1. Disinfection of equipment & environments commercial only	10](#_Toc236683596)

[eFigure 2. Entry/exit decontamination stations-vehicles commercial only	11](#_Toc236683597)

[eFigure 3. Entry/exit decontamination stations – humans commercial only	12](#_Toc236683598)

[eFigure 4. Personal hygiene practices commercial only	13](#_Toc236683599)

[eFigure 5. Personal protective equipment & dedicated apparel - contact transmission pathway – commercial only	14](#_Toc236683600)

[eFigure 6. Personal protective equipment & dedicated apparel – respiratory particles – commercial only	15](#_Toc236683601)

[eFigure 7. Quarantine & cross-farm contamination control commercial only	16](#_Toc236683602)

[eFigure 8. Information Resources in Biosecurity Practices commercial only	17](#_Toc236683603)

[eFigure 9. Waste & carcass management commercial only	18](#_Toc236683604)

[eFigure 10. Disinfection of equipment & environments - no high ROB	19](#_Toc236683605)

[eFigure 11. Entry/exit decontamination stations-vehicles - no high ROB	20](#_Toc236683606)

[eFigure 12. Entry/exit decontamination stations-humans - no high ROB	21](#_Toc236683607)

[eFigure 13. Personal hygiene practices - no high ROB	22](#_Toc236683608)

[eFigure 14. Personal protective equipment & dedicated apparel - contact transmission pathway - no high ROB	23](#_Toc236683609)

[eFigure 15. Personal protective equipment & dedicated apparel - respiratory particles/mixed - no high ROB	24](#_Toc236683610)

[eFigure 16. Quarantine & cross-farm contamination control – no high ROB	25](#_Toc236683611)

[eFigure 17. Information Resources in Biosecurity Practices – no high ROB	26](#_Toc236683612)

[eFigure 18. Waste & carcass management - no high ROB	27](#_Toc236683613)

# e Table 1. Search Strategy

| Set | Search Statement |
| --- | --- |
| 1 | Influenza in Birds/ |
| 2 | (H5N1 or "Avian influenza" or "avian flu" or "bird flu" or "Influenza A" or "influenza A virus subtype H5N1" or "fowl plague" or H5Nx or H5N*).tw. |
| 3 | 1 or 2 |
| 4 | Food Contamination/ or Equipment Contamination/ |
| 5 | Occupational Exposure/ or Environmental Exposure/ |
| 6 | (farmer or Healthcare worker or Agriculture worker or Clinical worker or laboratory worker).tw. |
| 7 | (bird or "avian species" or poultry or fowl or chicken or animal or mammal or seal or cow or bovine or cat or feline or dog or canine or ferret or pig).tw. |
| 8 | (farm or pasture or "Agricultural land" or farmland or Ranch or Homestead or Cropland or "Dairy farm" or "Livestock farm" or "Poultry farm" or Barnyard or Fields or Agribusiness or "Livestock farms" or "Dairy farms" or "Poultry farms" or "Agro-industrial site" or "Beef cattle farms" or "Swine farm" or "Mixed farms" or Agrotourism farms" or urban farms" or "chicken coop" or hen house" or bird or "avian species" or poultry or fowl or chicken or animal or mammal or seal or cow or bovine or cat or feline or dog or canine or ferret or pig or "table-egg" or "layer").tw. |
| 9 | (Contamination or exposure or contact or handling or travel or ingestion or consumption or interaction or fomite or direct contact or close contact or transmission or contagion or spread or communicability or human-to-human or animal-to-human or animal-to-animal or fomite-to-human or fomite-to-animal).tw. |
| 10 | Behavioral Risk Factor Surveillance System/ |
| 11 | 4 or 5 or 9 or 10 |
| 12 | 6 or 7 or 8 |
| 13 | Personal Protective Equipment/ |
| 14 | ("personal protective equipment" or PPE or mask* or respirator* or "N95" or FF? or FP3 or PAP or "powered air purifying respirator*" or goggle* or "face shield*" or "eye protection" or glove* or "protective clothing" or gown* or coverall* or "shoe cover*" or boot* or apron* or hood* or "head cover*" or hand hygiene" or "hand washing" or "hand sanit*" or "alcohol-based hand rub" or "soap and water" or "respiratory hygiene" or "cough etiquette" or "contact precaution*" or "droplet precaution*" or "airborne precaution*" or "standard precaution*" or "infection control" or "infection control" or "social distancing" or "physical distancing" or "avoid* contact" or "avoid exposure" or quarantine or isolation or "self-isolation" or "stay at home" or "risk communication" or "health education" or "preventive behaviour*" or "protective behaviour*" or "occupational safety" or "biosecurity measure*" or farm biosecurity" or biosecurity" or decontaminat* or disinfect* or clean*).tw. |
| 15 | Infection Control/ |
| 16 | Communicable Disease Control/ |
| 17 | 13 or 14 or 15 or 16 |
| 18 | 3 and 11 and 12 and 17 |
| 19 | H5N*.tw. |
| 20 | 18 and 19 |
| 21 | 3 and 12 and 17 |

# eTable 2. Characteristics of the included studies

| Study | Study type | Poultry type | Outbreak year | Sample Size | Setting | Country | avian subtype | Case Identification | Confirmation of non-infectious status at baseline (cohort studies only) | Sample to confirm case | Test to confirm case | Control Selection | Control Identification | Matched Controls | Sample to confirm non-infection status in controls | Test to confirm non-infectious status in controls |
| --- | --- | --- | --- | --- | --- | --- | --- | --- | --- | --- | --- | --- | --- | --- | --- | --- |
| Biswas 2009 (backyard) | case-control | chicken | March 2007 to November 2007 | 100 | backyard | Bangladesh | HPAI H5N1 | farms with recorded outbreaks | n/a; case control study | 2 chickens in farms with high chicken mortality | tracheal RT-PCR | 3 control farms for each case | farms in villages without outbreak | area (within 1–10 km) | noninfected status not confirmed | noninfected status not confirmed |
| Biswas 2009 (commercial) | case-control | chicken | January to September 2007 | 132 | commercial (broiler and layer) | Bangladesh | HPAI H5N1 | farms with recorded outbreaks in the National Reference Laboratory for Avian Influenza | n/a; case control study | diseased and dead chickens | tracheal RT-PCR (>1 positive) | 3 control farms for each case | farms without recorded outbreaks | area (within 1–10 km), production system | noninfected status not confirmed | noninfected status not confirmed |
| Biswas 2011 | case-control | chicken | February– April 2008 | 60 | small-scale commercial farms (FAO defined production system 3) | Bangladesh | HPAI H5N1 | farms with recorded outbreaks | n/a; case control study | dead chickens | tracheal RT-PCR (>1 positive) | 3 control farms for each case | farms with no clinical signs of illness | production system, area (within 1-10 km) | noninfected status not confirmed | noninfected status not confirmed |
| Chaudhry 2015 | case-control | chicken | May 2009 and January 2010 | 266 | commercial (broiler and layer) | Pakistan | H9N2 | regular surveillance, dead birds | n/a; case control study | dead birds | lung, intestine, trachea; hemagglutination inhibition test (HI) | 1:1; farms submitting to the same laboratory | lack of clinical signs of influenza and an average flock mortality of less than 3% | laboratory; timepoint (month of submission) | noninfected status not confirmed | noninfected status not confirmed |
| Chaudhry 2017 | Prospective cohort | chicken | November 2013 to February 2014 | 109 | commercial (broiler and layer) | Pakistan | H9N2 | regular surveillance, dead birds | not confirmed; all farms assumed negative due to absence of any influenza or influenzalike illness in the flock. | five to ten dead birds from total mortality on farm | trachea - Anigen Rapid AIVAg Detection Kit ; embryonated chicken eggs, hemagglutinin inhibition (HI) test. | n/a; cohort study | n/a; cohort study | n/a; cohort study | dead birds | trachea - Anigen Rapid AIVAg Detection Kit |
| Mehrabadi 2016 | cross-sectional | mixed (chicken, waterfowl, turkey, pigeon, quail, partridge, ostrich | September to December 2014 | 329 | backyard | Iran | H9N2 | random sampling, four backyard poultry farms per village | n/a; cross-sectional study | 11 birds/farm | blood sample (wind vein); Enzyme Linked Immunosorbent Assay (ELISA) A-Type-commercial kit | n/a; cross sectional | n/a; cross sectional | n/a; cross sectional | random sampling, four backyard poultry farms per village | blood sample (wind vein); Enzyme Linked Immunosorbent Assay (ELISA) A-Type-commercial kit |
| Fasina 2011 | case-control | unspecified | February 2006 to October 2007 | 115 | commercial and non-commercial | Nigeria | HPAI H5N1 | list of indected farms, stratified by state | n/a; case control study | serum sample; unspecified | unspecified virological tests | 2.59 controls for each case | farms with seronegative test results | area (same state); timepoint (sample collection time);flock size (<500 or >500) | regular surveillance; unspecified | serum samples; test not specified |
| Garber 2016 | case-control | chicken | May 2025 | 59 | commercial (table-egg layers) | USA | HPAI H5N2 | regular surveillance | n/a; case control study | unspecified | virologic or molecular detection; unspecified | 1:1 from surrounding area (16km) | regular surveillance | area; within 16 km of case farm | regular surveillance; unspecified | virologic or molecular detection; unspecified |
| Gompo 2020 | case-control | chicken and duck | March 2018 to April 2019 | 100 | commercial and non-commercial | Nepal | H9 | regular surveillance | n/a; case control study | unspecified | rapid antigen followed by PCR | 1:1 from surrounding area (3km) | farms tested negative for AI during outbreak | farm size and area (3km) | unspecified | PCR |
| Green 2023 | case-control | chicken | February through September 2022 | 40 | commercial (table-egg layers) | USA | HPAI H5N1 | regular surveillance | n/a; case control study | unspecified | RT-PCR | 1:1 from the same state | regular surveillance | area (state) | unspecified | PCR |
| Guinat 2020 | case-control | duck | winter 2016–2017 | 226 | commercial | France | HPAI H5N8 | regular surveillance | n/a; case control study | unspecified | at least one confirmed case by virus isolation or PCR | 1;1 within 10km | regular surveillance | area (within 10 km); production type | unspecified | virus isolation or PCR |
| Gupta 2022 | cross-sectional | chicken | February to April 2017 | 106 | commercial farms | Baglandesh | H5 and H9 | unspecified | n/a; cross-sectional study | blood sample: 1–3 ml of blood from the wing or jugular vein (healthy chicken), 9 chickens/layer farm; 8 chickens/broiler farm | ELISA and hemagglutination inhibition test | n/a; cross sectional | n/a; cross sectional | n/a cross sectional | blood sample: 1–3 ml of blood from the wing or jugular vein (healthy chicken), 9 chickens/layer farm; 8 chickens/broiler farm | ELISA and hemagglutination inhibition test |
| Henning 2009 | case-control | duck and chicken | December 2006 to January 2007 | 47 | small-holder non-commercialduck and chicken farms | Vietnam | HPAI H5N1 | farms with recorded outbreaks | n/a case control | sick birds | PCR | 2 controls for each case | no outbreak occurring on the farm 2 weeks before or 2 weeks after the first day of the outbreak case farm; crude mortality less than 5%. | the time of outbreak, farm location, species | sick birds | PCR |
| Islam 2024 (turkey) | cross-sectional study | turkey | March to August 2019 | 197 | commercial and non-commercial | Baglandesh | H5 (19%); H9 (17%) | 197 turkey farms across 9 districts (out of 64) selected based on turkey density and trading regions | n/a cross sectional | oropharyngeal and cloacal swabs; 4 random turkeys/farm on average (range 2-6 based on farm size), | rRT-PCR | n/a; cross sectional | n/a; cross sectional | n/a; cross sectional | oropharyngeal and cloacal swabs; 4 random turkeys/farm on average (range 2-6 based on farm size), | rRT-PCR assay |
| Islam 2024 (chicken) | cross-sectional study | chicken | January to July 2017 | 225 | commercial farms | Baglandesh | H5 (13%); H9 (14%) | random sampling, 225 commercial chicken farms in 6 different districts | n/a cross sectional | pooled fresh fecal sample (4 swabs/farm); cloacal and oropharyngeal swabs from dead or sick birds | rRT-PCR | n/a; cross sectional | n/a; cross sectional | n/a; cross sectional | pooled fresh fecal sample (4 swabs/farm); cloacal and oropharyngeal swabs from dead or sick birds | rRT-PCR |
| Jensen 2025 | case-control | chickens, turkeys, ducks, geese, pheasants,fowls | 5 cases from 2020/2021 season, 6 from 2021/2022 ; 7 from 2022/2023 | 31 | commercial farms | Denmark | HPAI | holdings with recorded outbreaks | n/a case control | unspecified | PCR followed by sequencing | 2 controls for each case | lack of clinical signs | size of the holding (<100, 101–10,000 or >10,000 birds); species,distance from coastal areas or wetlands (0–1500 m, 1501–3000 m, 3001–4500 m, 4501– 6000 m or 6001–7500 m) | noninfected status not confirmed | noninfected status not confirmed |
| Kim 2022 | case-control | poultry (unspecified) | March-April 2016; November 2016 - March 2017; February - April 2017; June 2017 | 288 | commercial and non-commercial | Korea | HPAI | farms with recorded outbreaks | n/a case control | unspecified | unspecified | 1:1, identified from the same database | farms without outbreaks | no mention | noninfected status not confirmed | noninfected status not confirmed |
| Kim 2018 | Case-control | broiler ducks | January 2014- April 2016 | 86 | commercial farms | Korea | HPAI H5N8 | regular surveillance, stratified by proportion of H5N8 cases | n/a case control | dead birds | RT-PCR | 1:1 from surrounding area (3km) | farms without outbreaks | area (within 3km) | unspecified | PCR |
| Kondo 2025 | case-control | layer farms (unspecified) | October 2022 - April 2023 | 84 | commercial farms | Japan | HPAI H5N1 | farms with reported outbreaks | n/a case control | unspecified | unspecified | Max of 2 controls per case farm (no eligible controls found for 19 of 58 cases) | random sampling, farms without recorded outbreaks within 5km | area (within 5km) | noninfected status not confirmed | noninfected status not confirmed |
| Lambrou 2020 | cross-sectional | chicken and turkey | July 2013-August 2018 | 150 | commercial farms | Nepal | HPAI | district farm registry unspecified | n/a cross sectional | self reported | n/a self reported | n/a; cross sectional | n/a; cross sectional | n/a; cross sectional | self reported | n/a self reported |
| Lohiniva 2013 | case-control | poultry (unspecified) | January 2009 - June 2009 | 56 | household poultry | Egypt | HPAI H5N1 | househould poultry farms with reported outbreaks | n/a case control | unspecified | unspecified | 2 household control farms selected for each case | the closest household reporting no human H5N1 cases or poultry H5N1 outbreaks | area (unspecified distance) | noninfected status not confirmed | noninfected status not confirmed |
| McQuiston 2005 | case-control | chicken and turkey | April 2002 | 350 | commercial farms | USA | LPAI H7N2 | regular surveillance, dead birds | n/a case control | dead birds (fresh samples) | ELISA and RT-PCR (fresh tracheal swab) | 1:1 (approx.) from the same state | regular surveiallance; remaining noninfected farms | species (chicken and turkey); area (1mile) | regular surveillance; dead birds | ELISA and RT-PCR (fresh tracheal swab) |
| Métras 2013 | case-control | mixed (predominally layer chicken) | April and May 2009 | 110 | commercial farms | Nigeria | HPAI H5N1 | regular surviellance; comvenience sampling, farms with confirmed outbreak; | n/a case control | sick and dead birds | PCR. Tracheal and cloacal swabs for live birds; tracheas, lungs, spleens, brains, hearts, intestines and intestinal contents for dead birds | 1:1 from the same state | regular surveillance; no reported outbreaks | area (state) | sick and dead birds | PCR. Tracheal and cloacal swabs for live birds; tracheas, lungs, spleens, brains, hearts, intestines and intestinal contents for dead birds |
| Mirzaie 2021 | prospective cohort study | chicken | July 2016 - November 2017 | 34 | commercial broiler farms | Iran | LPAI H9N2 | regular surveillance | farms that were PCR-positive at the initial day-3 sampling were excluded from the cohort to ensure all enrolled farms were initially disease-free. | sick birds sampled at days 3, 10, 20, 30, 40, and 50 of age | 60 tracheal/cloacal swab samples were collected per flock; PCR | n/a; cohort study | n/a; cohort study | n/a; cohort study | sick birds | tracheal/cloacal swabs; PCR |
| Nishiguchi 2007 | case-control | chicken | April to August 2005 | 73 | commercial layer farms | Japan | Low-Pathogenic H5N2 | farms with recorded outbreaks | n/a case control | unspecified | seropositivity testing (no other details) | 1:1 from surounding area (5km) | farms without recorded outbreaks | wthin the same movement restrction zone (5km of infected farm) | noninfected status not confirmed | noninfected status not confirmed |
| Osmani 2014 | case-control | chicken | 2011 | 265 | commercial layer farms | Bangladesh | HPAI H5N1 | random sampling of farms with reported outbreaks | n/a case control | sick birds | Antigen test (Anigen Rapid AIV Ag Test) and RT-PCR | 2 controls for each case within same area | farms without recorded outbreaks | area (within 5km of case farm); random sampling | noninfected status not confirmed | noninfected status not confirmed |
| Patyk 2023 | case control | turkey | 2022-2023 | 125 | commercial farms | USA | HPAI H5N1 | farms with recorded outbreaks | n/a case control | unspecified | RT-PCR, pathotyping assay, whole genome sequencing | 2-5 control farms per case (results show approx 1:1); same state | farms without recorded outbreaks; random sampling | area (state) | noninfected status not confirmed | noninfected status not confirmed |
| Paul 2011 | case-control | chicken | January 2004 and 31 July 2005. | 486 | backyard farms | Thailand | HPAI H5N1 | farms with recorded outbreaks | n/a case control | sick and dead birds | RT-PCR and virus isolation | 3-4 control farms per case farm | farms with no reported outbreaks or those with clinical suspicion but tested negative | no mention | sick birds | unspecified laboratory tests… otherwise noninfected status not confirmed |
| Subedi 2022 | case control | chicken | October 2019 to March 2020 | 102 | commercial and non-commercial | Nepal | LPAI H9 Subtype | regular surveillance | n/a case control | sick and dead birds | rapid antigen followed by PCR | one control for each case | regular surveillance of sick and dead birds | timepoint (sample submitted same week) | sick and dead birds | rapid antigent |
| Thompson 2025 | case control | turkey | January to October 2022 | 86 | commercial farms | USA | HPAI | unspecified | n/a case control | unspecified | unspecified | 2-5 controls per case in the same state randomly selected. | unspecified | no mention | unspecified | unspecified |
| Thompson 2008 | cross-sectional | ostrich | end of 2005 | 367 | commercial farms | South Africa | H5 | regular surveillance; all registered ostrich farms | n/a case control | 15-60 serum samples/farm | haemagglutination inhibition | n/a cross sectional | n/a cross sectional | n/a cross sectional | 15-60 serum samples/farm | haemagglutination inhibition |
| Tombari 2013 | Cross-sectional | chicken and turkey | October 2010 to May 2011 | 624 | commercial flocks | Tunisia | LPAI | unspecified; commercial flocks around Tunisia | n/a cross sectional | 20 blood samples/flock; 20 cloacal and tracheal samples/flock | cELISA and rRT-PCR | n/a cross sectional | n/a cross sectional | n/a cross sectional | 20 blood samples/flock; 20 cloacal and tracheal samples/flock | cELISA and rRT-PCR |
| Tran 2024 | case-control | unspecified | November 2021 to January 2022 | 150 | backyard premises | Vietnam | H5N6 and H5N8 | random sampling form list (national agriculture records) | n/a case control | sick poultry with 2 or more clinical signs | RT-PCR | households in same village with not sick birds | national agriculture records | area (village); poultry type; timepoint (2 weeks before to 2 weeks after outbreak) | sick poultry with 2 or more clinical signs | RT-PCR |
| Wakawa | case-control | poultry (unspecified) | 2006-2008 | 64 | commercial farms | Nigeria | HPAI H5N1 | random sampling from list of infected farms | n/a case control | unspecified | unspecified | 1 control for each case | random sampling from list of infected farms | no mention | unspecified | unspecified |
| Wang 2018 | cross-sectional | mixed (unspecified) | between May 2015 and May 2017. | 352 | backyard premises | China | H5, H7, H9 | stratified by city | n/a cross sectional | 15-30 samples/month/city; total of total of 4538 samples; feces (predominally), anal swabs, throat swabs, feather swabs, poultry cage swabs, drinking water samples, crib swabs, sewage samples, environmental surface swabs, and chopping board swabs | RT-PCR | n/a cross sectional | n/a cross sectional | n/a cross sectional | 15-30 samples/month/city; total of total of 4538 samples; feces (predominally), anal swabs, throat swabs, feather swabs, poultry cage swabs, drinking water samples, crib swabs, sewage samples, environmental surface swabs, and chopping board swabs | RT-PCR |
| Wells 2017 | Case-control | turkey | 2015 | 83 | commercial farms | USA | HPAI H5N2 | list of infected farms from unspecified independent turkey company | n/a case control | unspecified | rRT-PCR | 1 control for each case | list of non infected farms from unspecified independent turkey company | production stage | unspecified | rRT-PCR |
| Woo 2008 | case-control nested within cross-sectional | chicken and duck | Between November 2005 and March 2006 | 40 | commercial and non-commercial | Korea | LPAI H9N2 | random sampling stratified by bird type and production system; then positive farms with employees matched to negative farms with employees | n/a case control | 15 blood samples per flock | hemagglutination inhibition ( layers, broilers, broiler breeders), agar gel precipitation (AGP) test (domestic ducks). | 1 control for each case | random sampling stratified by bird type and production system; then positive farms with employees matched to negative farms with employees | number of hired employees, flock age, and flock size | 15 blood samples per flock | hemagglutination inhibition ( layers, broilers, broiler breeders), agar gel precipitation (AGP) test (domestic ducks). |
| Yari 2023 | cross-sectional study | chichen, turkey, quail, duck, ornamental birds | end of 2018 to 2019 | 128 | commercial farms | Iran | H5N1 | stratified sampling method based on the ratio of poultry breeding units in each district | n/a cross sectional | unspecified | unspecified | n/a; cross sectional | n/a; cross sectional | n/a; cross sectional | unspecified | unspecified |
| Yoo 2022 | Case-control | chicken | November 16, 2016, to March 2, 2017. | 243 | commercial (layer) farms | Republic of Korea | HPAI H5N6 | national surveillance | n/a case control | unspecified | unspecified HPAI negative test | 1 control for each case | random sampling, national surveillance, stratified by municipality | no mention | unspecified | unspecified HPAI negative test |

# eTable 3. Risk of bias

# eFigure 1. Mapping table linking extracted exposure variable to the pooled analytical category

# eFigure 2. Disinfection of equipment & environments commercial only

# eFigure 3. Entry/exit decontamination stations-vehicles commercial only

# eFigure 4. Entry/exit decontamination stations – humans commercial only

# eFigure 5. Personal hygiene practices commercial only

# eFigure 6. Personal protective equipment & dedicated apparel - contact transmission pathway – commercial only

# eFigure 7. Personal protective equipment & dedicated apparel – respiratory particles – commercial only

# eFigure 8. Quarantine & cross-farm contamination control commercial only

# eFigure 9. Information Resources in Biosecurity Practices commercial only

# eFigure 10. Waste & carcass management commercial only

# eFigure 11. Disinfection of equipment & environments - no high ROB

# eFigure 12. Entry/exit decontamination stations-vehicles - no high ROB

# eFigure 13. Entry/exit decontamination stations-humans - no high ROB

# eFigure 14. **Personal hygiene practices - no high ROB**

# eFigure 15. Personal protective equipment & dedicated apparel - contact transmission pathway - no high ROB

# eFigure 16. Personal protective equipment & dedicated apparel - respiratory particles/mixed - no high ROB

# eFigure 17. Quarantine & cross-farm contamination control – no high ROB

# eFigure 18. Information Resources in Biosecurity Practices – no high ROB

# eFigure 19. Waste & carcass management - no high ROB
